# Liver injury in COVID-19: prevalence and its associated factors in Nepal – A retrospective cross-sectional study

**DOI:** 10.1097/MS9.0000000000000767

**Published:** 2023-05-03

**Authors:** Bibek Rajbhandari, Suman Pant, Achyut B. Hamal, Ashish Thapa, Apurba Shrestha, Saurav Shrestha, Anil Shrestha, Niranjan Panta, Udesh Pandey, Mukul Upadhyay Nepal, Olita Shilpakar

**Affiliations:** a Department of Emergency Medicine and General Practice, Nepal Police Hospital; b Nepal Health Research Council; c Department of Hepatobiliary, Nepal Police Hospital; d Department of Emergency Medicine and General Practice; e Department of Medicine; f Department of Anesthesia; g Department of Operation and Data Management, Nepal A.P.F. Hospital; h Department of Health Services, Ministry of Health and Population; i Department of Emergency Medicine and General Practice, Greencity Hospital; j National Society for Emergency and Disaster Risk Management (NSEDRM); k Department of Emergency, Tribhuvan University Teaching Hospital, Kathmandu, Nepal

**Keywords:** COVID-19, liver injury, prevalence, Nepal

## Abstract

Liver impairment has been reported as a common clinical manifestation in patients with Severe Acute Respiratory Syndrome Coronavirus 2 infection, with varying degrees of severity ranging from a mild elevation of liver enzymes to acute liver failure. However, the prevalence and clinical significance of liver injury in Coronavirus Disease 2019 (COVID-19) patients, particularly in low- and middle-income countries such as Nepal, remains poorly understood. To investigate the hepatic injury related to this disease and its clinical significance, the authors conducted a retrospective study that included adult patients with COVID-19 infection in security hospitals of Nepal.

**Methodology:**

The authors performed a retrospective chart review on 1007 COVID-19-positive patients who underwent a liver function test during their admission in two COVID-19 dedicated hospitals in Nepal from April 2021 to January 2022. The data were collected and entered into Microsoft Excel before being exported to SPSS version 24 for analysis. Univariate and multivariate logistic regression were used to determine factors associated with liver injury. We reported adjusted odds ratios (aOR) with 95% CI.

**Results:**

Of the total, 549 (54.5%) patients had an acute liver injury. Among 549 patients, 68.1% were mild, 27.9% were moderate, and 5.0% were severe. Out of 1007 patients, 1.4% had cholestatic liver injury. Most patients with mild, moderate, and severe liver injury had greater than or equal to 10 C-reactive proteins (CRP). In multivariate logistic regression, sex, and CRP were significantly associated with the presence of liver injury. Males had 1.78 times higher odds of having a liver injury compared to females (aOR:1.78; 95% CI: 1.37–2.30, *P*-value:<0.001). Similarly, patients who had CRP greater than 10 had higher odds of having liver injury compared to those who had CRP less than 10 (aOR: 1.84; 95% CI: 1.41–2.39; *P*-value: <0.001).

**Conclusions:**

The present study reveals that COVID-19 infection is commonly associated with mild increased liver enzymes. However, the likelihood of developing acute liver injury was found to be higher in patients with an inflammatory state indicated by CRP levels greater than 10. Furthermore, the study highlights the sex-based difference in the prevalence of liver injury, with males demonstrating a higher predisposition.

## Introduction

Highlights

- Out of 1007 patients, 549 (54.5%) patients had acute liver injury. Among 549 patients, 68.1% were mild, 27.9% were moderate, and 5.0% were severe.
- The population with elevated C-reactive protein (>10) had a higher risk of developing acute liver damage.
- Males had 1.78 times higher odds of having a liver injury compared to females (aOR:1.78; 95% CI:1.37–2.30, *P*-value:<0.001).
- Rather than relying just on the patient’s history, a thorough physical examination and investigation would have provided more solid evidence in ruling out chronic liver disease.

In December 2019, a novel coronavirus severe acute respiratory syndrome coronavirus 2 (SARS-CoV-2) infection was discovered in the Hubei province of Wuhan, China, and it has since spread to 220 countries resulting in more than 200 million confirmed cases and 4 million deaths as of August 2021^1^. Coronavirus disease 2019 (COVID-19) is the term given to the sickness produced by the virus SARS-CoV-2. The virus can produce a short-term respiratory infection, but it can also cause severe alveolar destruction and respiratory failure^2^. The gastrointestinal system and the liver, in particular, have been discovered to be damaged, even though the presenting symptoms are mostly respiratory^3^. SARS has been observed to cause damage to hepatocytes in addition to alveoli by means of the angiotensin-converting enzyme 2 receptor, which is expressed extensively within the liver^4^. While electron microscopy was unable to detect viral particles, coronavirus presence in liver tissues was detected through reverse transcription polymerase chain reaction^5^. Several types of vaccinations have been developed to combat the COVID-19 pandemic^6–8^. In addition to the well-documented benefits of vaccination in mitigating the spread of COVID-19^9^, recent studies have reported instances of liver injury among individuals who received the COVID-19 vaccine^10^.

A significant number of COVID-19 patients experience abnormal liver function, as demonstrated in a study comprising 5700 cases from New York, where more than half exhibited abnormal liver enzymes^11^. Further research has corroborated these findings through numerous articles documenting elevated levels of aminotransferase in COVID-19 patients^12–19^. A recent systematic review and meta-analysis on liver function test (LFT) abnormalities found that there was a pooled increase of aspartate amino transferase (AST) and alanine amino transferase (ALT) in approximately one-third to one-fourth of cases, respectively^20^. During the progression of the illness, a significant percentage ranging from 14 to 53 exhibited abnormal levels of ALT and AST^21–23^. The synthesis activity within the liver is also impaired during infection with COVID-19; this was evidenced by prothrombin time prolongation being most pronounced among individuals experiencing digestive symptoms alongside disrupted hepatic functioning overall^24^.

Previous research has identified several potential mechanisms for liver damage in severe COVID-19 cases, including direct viral injury, immune-mediated damage, and drug-induced injury^25–28^. These studies also highlight the challenges in diagnosing liver injury in COVID-19 patients, especially those with pre-existing liver conditions. Liver injury is a significant complication in severe cases of COVID-19, and further research is necessary to comprehend its underlying mechanisms and develop effective management approaches for affected patients.

Although liver abnormalities have been reported in adult patients with COVID-19 infection, the prevalence, and their link with outcomes have not yet been investigated in Nepal. This knowledge gap is particularly relevant because Nepal is a low-income country with limited resources and a high burden of infectious diseases. Therefore, understanding the pattern of liver injury in COVID-19 patients in Nepal is crucial for developing effective management strategies and improving patient outcomes. To address this research gap, we aimed to investigate the prevalence of liver injury and its associated factors in COVID-19 patients.

## Materials and methodology

### Study design and setting

We performed a retrospective paper and electronic chart review of COVID-19-positive patients who did the LFT in two security hospitals of Nepal. All patients with LFT between April 2021 to January 2022 were included.

### Study populations

The study enrolled all patients admitted within a specified timeframe, and those with pre-existing liver disease indicated by regular alcohol consumption, chronic hepatitis, long-term medication use, and obesity were excluded using history records. Missing data were gathered through phone calls or physical follow-ups. Prior to admission, all COVID-19-positive patients underwent routine laboratory investigations as per hospital criteria, including LFTs. The LFTs measured AST and ALT levels, and a moderate level of liver injury was defined as AST and ALT levels exceeding two but less than five times the Upper Level of Normal Limit (ULN). Data from 1007 patients were extracted and analyzed in this study.

### Extracted variables

The following variables were extracted from the record of the hospitals:

1. **Age:** It refers to the chronological age of the respondents.
2. **Sex:** It refers to the biological sex; males and females.
3. **C-reactive protein:** C-reactive protein (CRP) is an acute phase protein whose serum levels rise in response to infections and non-infectious inflammatory processes in a nonspecific manner. It is produced in the liver and is normally found in trace amounts in the blood. CRP levels may rise above normal within four hours after an acute event in various disease states resulting in tissue injury, infection, or inflammation. Understanding the amount of CRP produced can be advantageous for the diagnosis, treatment, and observation of inflammatory diseases. CRP increases are nonspecific, and they should not be interpreted without a full clinical history^29^. The CRP-Latex Test is a prompt slide agglutination procedure that has been adapted from the latex fixation method and is utilized to directly detect and semi-quantitatively assess CRP levels present in serum^30^.
4. **Transaminase:** ALT and AST, two types of serum aminotransferases formerly referred to as glutamic-oxalacetic transaminase (SGOT) and glutamic-pyruvic transaminase (SGPT), respectively, are employed for the diagnosis and evaluation of liver disease. ALT has been considered a dependable and sensitive indicator of liver dysfunction based on its concentration in the blood^31^. In acute viral hepatitis or hepatotoxic drug reactions, both AST and ALT levels rise proportionately. Conversely, chronic hepatitis or cirrhosis is characterized by higher serum AST than ALT due to hepatic cell necrosis causing the mitochondrial release of AST^32^. Transaminase-catalyzed reaction deplete nicotinamide adenine dinucleotide leading to the altered photometric intensity measured at 340 nm using kinetic UV technology for transaminase assessment purposes^33^.
5. **Alkaline phosphatase:** Alkaline phosphatase (ALP) is a membrane-bound metalloenzyme made up of many isoenzymes. A rise in the ALP liver isotype indicates the presence of cholestasis^34^. A kinetic approach is used to measure the amount of ALP by employing the enzyme’s substrate, p-nitrophenyl phosphate, and the rate at which the colored substrate, p-nitrophenol, is produced during the reaction^35^.
6. **Diagnosis of acute liver injury:** In this study, we have defined two distinct patterns of liver injuries, namely cholestatic and hepatocellular. Based on the degree of ALT elevation, the severity of the liver injury was classified as mild (2 times ULN), moderate (2–5 times the ULN), or severe (>5 times ULN)^36^. Additionally, the cholestatic pattern was identified by ALP levels exceeding twice the upper limit of normal^37^.

### Data analysis

To determine the prevalence of liver injury, we assessed the levels of SGPT/SGOT and ALP in the patients. Furthermore, we collected general characteristics information such as age, sex, and CRP, which were recorded in Ms. Excel and later exported to SPSS version 24 for statistical analysis. We presented categorical variables as percentages and frequencies, whereas numerical data were expressed as means and standard deviations. To identify factors associated with liver injury at a significance level of 0.05, we employed univariate and multivariate logistic regression analysis. The results were presented as crude odds ratio and adjusted odds ratio (aOR) with a 95% CI.

### Ethical considerations

We obtained ethical clearance from the Ethical Review Board (ERB), Nepal Health Research Council (NHRC). We also received permission letters from the respective hospitals.

This study is in line with Strengthening the reporting of cohort studies in surgery (STROCSS) Criteria^38^.

## Results

### General characteristics of the patients

Table 1 summarizes the general characteristics of patients. The mean age of the patients was 42.36±17.27 years. The majority of the patients were male (60.7%) and had CRP greater than or equal to 10 (55.2%).

**Table 1:** General characteristics (*n*=1007)

Characteristics	*n* (%)
Sex	
Male	611 (60.7)
Female	396 (39.3)
Age in years (Mean, SD)	42.36 (17.27)
CRP	
<10	451 (44.8)
≥10	556 (55.2)

n, frequency %: percentage.

### Prevalence of liver injury

Table 2 presents the prevalence of acute liver injury based on the SGPT/SGOT report. Of the total, 549 (54.5%) patients had acute liver injury. Among 549 patients, 68.1% were mild, 27.9% were moderate, and 5.0% were severe.

**Table 2:** Prevalence of acute liver injury in COVID-19 patients (n=1007)

Characteristics	*n* (%)
Presence of Liver Injury	
No	458 (45.5)
Yes	549 (54.5)
Severity of Liver Injury (*n*=549)	
Mild	374 (68.1)
Moderate	153 (27.9)
Severe	22 (4.0)

*n, frequency %: percentage.*

Table 3 presents the presence of liver injury based on the ALP. Out of 1007 patients, 1.4% had cholestatic liver injury.

**Table 3:** Prevalence of cholestatic liver injury (*n*=1007)

Liver Injury	*n* (%)
No	993 (98.6)
Yes	14 (1.4)

*n, frequency %: percentage.*

Table 4 presents the prevalence of liver injury in relation to CRP and sex. Most patients with mild, moderate, and severe liver injury had greater than or equal to 10 CRP. Males had a high prevalence of liver injury in comparison with females.

**Table 4:** Prevalence of liver injury according to gender and CRP (*n*=1007)

Characteristics	Normal *n* (%)	Mild *n* (%)	Moderatex *n* (%)	Severe *n* (%)
CRP				
<10	247 (53.9)	146 (39.0)	50 (32.7)	8 (36.4)
≥10	211 (46.1)	228 (61.0)	103 (67.3)	14 (63.6)
Gender				
Male	242 (52.8)	241 (64.4)	108 (70.6)	20 (90.9)
Female	216 (47.2)	133 (35.6)	45 (29.4)	2 (9.1)

*n, frequency %: percentage.*

### Association between liver injury and general characteristics

Table 5 presents an association between liver injury and other characteristics. In multivariate logistic regression, sex, age, and CRP were significantly associated with the presence of liver injury. Males had 1.78 times higher odds of having liver injury compared to females (aOR:1.78; 95% CI:1.37–2.30, *P*-value:<0.001) after adjusting general characteristics. Similarly, patients who had CRP greater than 10 had higher odds of having liver injury compared to those who had CRP less than 10 (aOR: 1.84; 95% CI: 1.41–2.39; *P*-value: <0.001) after adjusting general characteristics.

**Table 5:** Association between liver injury and general characteristics

Variables	cOR (95% CI)	*P*	aOR (95%CI)^*^	*P*
Sex				
Female	1		1	
Male	1.83 (1.41–2.36)	<0.001^*^	1.78 (1.37–2.30)	<0.001^*^
Age (Mean, SD)	1.01 (1.01–1.02)	0.002^*^	1.00 (1.00–1.01)	0.05
CRP				
<10	1		1	
≥10	1.98 (1.54–2.55)	<0.001^*^	1.84 (1.41–2.39)	<0.001^*^

* *P-value: <0.05.*

*Adjusted variables, Sex, Age, and CRP; aOR, adjusted odds ratio; CI, confidence interval; cOR, crude Odds Ratio.*

## Discussion

COVID-19 is a global pandemic caused by the SARS-CoV-2 virus. COVID-19 causes minor flu-like symptoms in the majority of people, but in extreme cases, it can cause considerable damage to the lungs and other essential organs, leading to acute respiratory distress syndrome, multiple organ failures, and even death^39^. Although the liver has been implicated in adult patients with COVID-19 infection, the prevalence of liver abnormalities and its link with outcomes have not yet been investigated in Nepal. In this context, we conducted a study to find out the prevalence of liver injury among COVID-19 patients in tertiary hospitals.

In this study, the mean age of the patients was 42.36±17.27 years. The majority of patients were male (60.7%). The total prevalence of acute liver injury was found to be 54.5% (549) based on transaminase liver enzymes. Among those 549 patients, 68.1% were in mild status whereas moderate and severe were 28 and 4%, respectively. The prevalence was more in males than females. In a population where CRP was in an inflammatory status (>10), the chances of acute liver injuries increased to a higher grade in comparison with that of a noninflammatory CRP status.

In line with our findings, the results of previous research indicate that COVID-19 patients experienced a significant rise in CRP levels, with an average range of 20–50  mg/l^21,40^. Studies have shown that elevated levels of CRP were observed in up to 86% of severe COVID-19 cases^21,41,42^. Earlier research findings suggest that increased levels of inflammatory cytokines in the bloodstream are associated with damage to the liver and lungs in cases of COVID-19 infection. CRP is a liver-produced protein that rises in the blood in response to inflammation, stimulated by factors released by macrophages and T cells^43^. According to the evidence, COVID-19 induces a substantial increase in CRP levels due to the resulting inflammatory response. One potential explanation for this rise is the excessive production of pro-inflammatory cytokines that combat the virus^44,45^. However, if this system becomes overly active, it can cause harm to hepatic tissues, resulting in liver injury. This study highlights the significant impact of COVID-19 on the liver and how high levels of CRP can increase the chances of acute liver injuries. Therefore, it is crucial to closely monitor liver enzymes and CRP levels in COVID-19 patients, especially those with pre-existing liver conditions.

Our study had concurrent results as by Xie *et al* ^46^. Where males were more prone to liver injury than females, the cause might be the high-risk behavior pattern of males like consumption of alcohol and acquiring chronic hepatitis due to viral infection in the outgoing population. Healthcare professionals must take these findings into account and implement gender-specific interventions to reduce the risk of liver injury in male COVID-19 patients, such as encouraging them to reduce alcohol consumption and promoting vaccination against viral hepatitis. While carrying out multivariate regression, age did not exhibit a significant association with liver injury in COVID-19 patients. However, previous research has indicated that advanced age categories are at risk of severe acute liver disease^47,48^. This could be due to aging, increasing vulnerability toward the fibrotic response and greater susceptibility towards acute liver damage.

We excluded the population with pre-existing liver disease in our study but a multicentric study conducted in the United States showed that liver injury was more in one with pre-existing liver disease^49^. In two of the studies conducted in Wuhan; China, among severe and mild patients, there were significant differences in liver biochemical indexes that were equivalent to our result^15,21^. In a descriptive study done by Chen, the majority of patients had mild liver enzyme dysfunction, which was similar to what we discovered^21^.

The pathogenesis of liver damage in COVID-19 infection remains incompletely understood despite the existence of several proposed theories. It is essential to acknowledge that the occurrence of a liver injury can significantly impact a patient’s prognosis. To mitigate the risk of liver damage during an illness, several preventative measures can be employed. It is imperative to conduct a thorough assessment of patients to ascertain the presence of liver disease and make any necessary adjustments before commencing therapy. This should be accomplished not only through history-taking but also through physical examination and diagnostic investigations, which were not included in our study. It is vital to maintain ongoing monitoring of liver function throughout the course of COVID-19 infection, encompassing diagnosis, treatment, and monitoring stages to facilitate prompt intervention if warranted. Such an approach ensures that any adverse effects on liver function are detected early, enabling the timely implementation of necessary corrective measures. Hence, continuous monitoring of liver function is essential in the management of COVID-19 patients, ensuring optimal patient outcomes.

### Strength and limitation

To the best of our knowledge, this is the first report describing detailed liver pathological manifestations for hospitalized patients with COVID-19 in Nepal. Specifically, 54.5% of the COVID-19 patients in our study were found to have an acute liver injury. Our results suggest that the population with elevated CRP (>10) had a higher risk of developing acute liver damage.

There are a few limitations in this study. First, the exclusion of the chronic liver disease would have been more reliable if it had been done by physical examination and investigation rather than history taking only. Due to the exploratory nature of the study, which was not driven by formal hypotheses, the sample size calculation was waived. Instead, we hope that the findings presented here will encourage a larger cohort study or potentially some randomized controlled trials. Third, this is a retrospective study. The data in this study permit a preliminary assessment of the clinical course and outcomes of COVID-19 patients with liver injury. Further studies are still needed.

## Conclusions

In conclusion, increased liver enzymes were mild in the COVID-19 infection. When evaluating patients clinically, it is important to differentiate between the development of irregular liver function upon initial diagnosis and the appearance of abnormal liver function subsequent to treatment. The study findings also suggest that clinicians should consider monitoring liver function in patients with COVID-19, especially in males, and those with elevated CRP levels. Further research is needed to explore the underlying mechanisms of liver injury in COVID-19 patients and to develop effective interventions to prevent or mitigate liver injury in this patient population. Overall, this study provides important insights into the prevalence and risk factors associated with liver injury in COVID-19 patients, which has important clinical implications for the management of this disease.

## Ethical approval

The approval for this study was taken from the Ethical Review Board (Ref no: 1137).

## Consent

NA.

## Source of funding

This study was funded by the Nepal Health Research Council (Ref no. 20211009).

## Authors’ contribution

B.R., A.H., and S.P.: conceptualized the study; A.T., A.S., and S.S.: cleaned the data; B.R., A.S., N.P., and U.P.: analyzed the data; B.R., A.H., S.P., and O.S.: worked on methodology; A.T., B.R., S.S., and M.U.N.: worked on discussion, reviewed and edited; B.R. made the first draft; A.H. and S.P. supervised the study. All authors reviewed and agreed on the final version of the manuscript.

## Conflicts of interest disclosure

None.

## Research registration unique identifying number (UIN)

1. UIN: researchregistry8863.
2. https://www.researchregistry.com/browse-theregistry#home/registrationdetails/643e5b2eec2601002754e325/.

## Guarantor

Suman Pant.

## Data availability statement

Available upon reasonable request.

## Provenance and peer review

Not commissioned, externally peer-reviewed.
